# Supplementary material for: A new cubic transmuted power-function distribution: Properties, inference, and applications
Source: PLoS One. 2023 Feb 6;18(2):e0281419. doi: 10.1371/journal.pone.0281419 (PMC9901817; doi:10.1371/journal.pone.0281419)
Supplement: Attachment — Submitted filename: Response letter_R1.pdf [file pone.0281419.s001.pdf]

Dear Professor Editor,

Enclosed herewith are the pdf of the revised version of our paper entitled "*A New Cubic Transmuted Power-Function Distribution: Properties, Inference, and Applications*", which we hope you will find now satisfactory for publication in PloS One.

First, we would like to thank the Editor and the two reviewers for very constructive comments. In the revised version all suggestions and comments have been taken into account and addressed. All corrections and modifications are incorporated in the revised version and highlighted in RED color.

We now answer the comments made by the editor and reviewers in the order they appeared in the reports.

Additional requirements:

1. Please ensure that your manuscript meets PLOS ONE's style requirements, including those for file naming. The PLOS ONE style templates can be found at https://journals.plos.org/plosone/s/file?id=wjVg/PLOSOne_formatting_sample_main_body.pdf
and https://journals.plos.org/plosone/s/file?id=ba62/PLOSOne_formatting_sample_title_authors_affiliations.pdf

Answer: We did our best to ensure that the paper is prepared according to PloS ONE style.

2. In your Data Availability statement, you have not specified where the minimal data set underlying the results described in your manuscript can be found. PLOS defines a study's minimal data set as the underlying data used to reach the conclusions drawn in the manuscript and any additional data required to replicate the reported study findings in their entirety. All PLOS journals require that the minimal data set be made fully available. For more information about our data policy, please see http://journals.plos.org/plosone/s/data-availability.

""Upon re-submitting your revised manuscript, please upload your study's minimal underlying data set as either Supporting Information files or to a stable, public repository and include the relevant URLs, DOIs, or accession numbers within your revised cover letter. For a list of acceptable repositories, please see http://journals.plos.org/plosone/s/data-availability#loc-recommended-repositories. Any potentially identifying patient information must be fully anonymized.

Important: If there are ethical or legal restrictions to sharing your data publicly, please explain these restrictions in detail. Please see our guidelines for more information on what we consider unacceptable restrictions to publicly sharing data:

http://journals.plos.org/plosone/s/data-availability#loc-unacceptable-data-access-restrictions. Note that it is not acceptable for the authors to be the sole named individuals responsible for ensuring data access.

We will update your Data Availability statement to reflect the information you provide in your cover letter.

Answer: This is not applicable in our case. The data set is already mentioned in the manuscript.

3. Please ensure that you refer to Figure 5 in your text as, if accepted, production will need this reference to link the reader to the figure.

Answer: Thank you. This typo is corrected.

Additional Editor Comments:

The paper is well written with publishable contents. However, some points have been noticed by reviewers, where the novelty of the paper needs to be further highlighted. The motivation is not clear and the impact of parameter selection should be discussed and analysed carefully.

Answer: Many thanks for these comments. We have addressed each and every comment raised by the two reviewers. All corrections have been incorporated in RED color.

Reviewers' Comments to the Authors:

Reviewer #1: The authors, in this paper, proposed a new three-parameter cubic transmuted power distribution for which some mathematical properties are derived. I have some major concerns about this paper. Please rearrange the paper using following comments.

1. The numerical technique is not properly discussed. Which numerical method or technique is used to attain the numerical solution of mathematical model.

Answer: Thanks for this comment. The R software is used to obtain simulation results and the empirical results. We mentioned this in Sections 5 and 6.

2. Abstract does not describe the major theme of the paper.

Answer: Thank you. The abstract is improved.

3. You have demonstrated real data sets. Please elaborate their impacts on parameters.

Answer: The estimates of the parameters in the application show a good fit to the electronic devices data, as shown in Figure 4.

4. How can be the performance of proposed estimators measured.

Answer: Thanks for this comment. The performance of the proposed estimators can be explored and measured using simulation results through calculating some measures such as the average biases (AB) and mean square errors (MSE) for all studied cases as shown in Tables 2-6.

5. What are the main features of new cubic transmuted-power function. Discuss in conclusion section.

Answer: Thanks for this comment. It is mentioned in the conclusion section.

6. What is new in the proposed mathematical model and why it is considered.

Answer: The idea behind this construction is simple and it aims to obtain more flexible models that adapt to empirical data distributions. It is not easy to find pdf and cdf that are shaped like a bathtub and the NCTPF model has that property.

Reviewer #2: Overall, the manuscript is technically sound. However, the writing in the abstract, introduction and conclusion needs some work in order to reach an acceptable level for publication in this journal. Some examples:

1. In the abstract, the authors mention that some mathematical properties are derived. The properties should be explicitly mentioned for many reasons.

Answer: Thanks for your comment. We have added them in the abstract.

2. Page 2: Middle paragraph starts with "Researchers mention..." This is not good writing. The authors should completely rewrite this paragraph.

Answer: Thanks for careful reading. We have corrected them.

3. In the conclusion, the first paragraph starts with "In this study..." Throughout the manuscript, the authors refer to it as a paper, study etc. These are informal terms and need some polishing.

Answer: Thank you. This typo is corrected.
